# EHMTI-0051. Prevalence of venous sinus stenosis in pseudotumor cereberi (PTC) using digital subtraction angiography (DSA)

**DOI:** 10.1186/1129-2377-15-S1-C20

**Published:** 2014-09-18

**Authors:** M Hamdy Ibrahim

**Affiliations:** 1 Neurology, Gulf Medical University GMU and Hospital, Ajman, United Arab Emirates

## Objectives

To study the prevalence of intracranial venous stenosis in Pseudotumor cereberi patients.

## Patients and methods

Thirty patients diagnosed as PTC according to Dandy criteria. All underwent general and neurological assessment. Radiological assessment included CT scan brain +/- MRI brain without contrast, MRV. All underwent digital subtraction cerebral Angiography (DSA) (venous phase) to confirm the validity of filing gaps seen at the level of MRV.

## Results

MRV brain showed that 24 patients (80%) showed filling gaps. Digital subtraction cerebral angiography (venous phase) showed 9 patients (30%) had stenosis in their dural sinuses. MRV showed to be a good screening tool since it had 100% sensitivity and negative predictive value. However, since it has a moderate specificity (62%) with a positive predictive value (PPV) of only 35%, then lesions detected should be confirmed with digital subtraction cerebral angiography (venous phase) particularly those involving the transverse and sigmoid sinus.

## Conclusion

Studying the intracranial venous system in patients with PTC is an important step in understanding the pathophysiology of the disease. Detection of venous sinus stenosis opens the way to a novel therapeutic option for refractory patients like venous sinus stenting.

No conflict of interest.

